# Three alleles in the *pat-3* locus of *Caenorhabditis elegans*: mutations in the membrane-distal NPxY phosphotyrosine motif

**DOI:** 10.17912/micropub.biology.000291

**Published:** 2020-08-15

**Authors:** Jacob Hanna, Shiva Ramani, Teja Williams, Ryan Anaya, Neil Campion, Evan Lopez, Raj Williams, Joe McIntire, Nicholas Tran, Victoria Reyna, Jingmei Zeng, Shailyn Miller, Amar Pancar, Zhongqiang Qiu, Myeongwoo Lee

**Affiliations:** 1 Department of Biology, Baylor University, Waco, Texas 76798, U.S.A.

**Figure 1:**
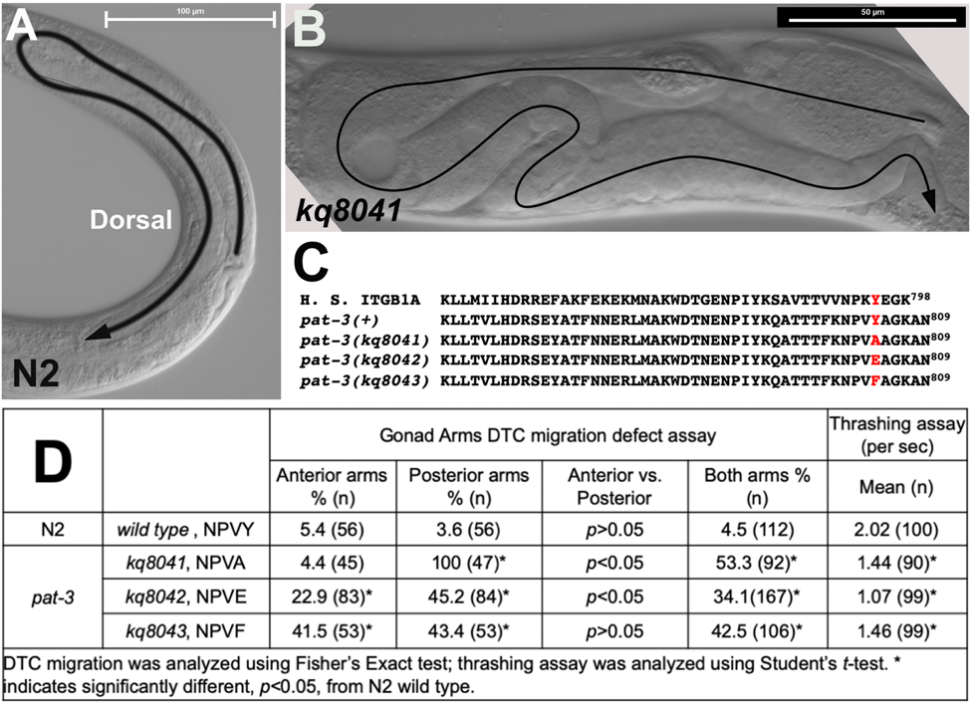
Characterization of PAT-3 membrane distal NPxY phospho-tyrosine motif. (A) N2 hermaphrodite gonad. Arrowhead and path indicate distal tip cell (DTC) migration. Bar = 100 μm; (B) A *pat-3(kq8041)*, Y804A, gonad showing migration defect. The gonad arm made extra turns. Arrowhead and path indicate DTC migration. Bar = 50 μm; (C) Protein sequence of wild type and mutant PAT-3 cytoplasmic tail was compared to human β1 integrin. Reds are the tyrosine and mutant residues in membrane distal NPxY; D. Gonad migration and motility analyses of *pat-3* mutants.

## Description

Integrin is a heterodimeric cell surface receptor for extracellular matrix proteins. *C. elegans* has two α integrin and one β integrin subunit. The β integrin PAT-3 contains two NPxY phospho-tyrosine motifs in the cytoplasmic domain (Figure 1C). The NPxY motif is known for interacting with talin and kindlins and plays essential roles in the bidirectional signaling of integrins (Hynes 2002). To investigate the role of tyrosine phosphorylation in the NPxY motifs, we mutated the tyrosine to different amino acids to mimic the physiological modifications. In this study, the membrane-distal NPxY was studied using genome editing with the CRISPR-Cas9 ribonucleoprotein complex system (Dickinson and Goldstein 2016). The membrane distal ^801^NPVY^804^ was engineered to three different forms, such as NPVF^804 ^(phenylalanine), NPVA^804^ (alanine), or NPVE^804^ (glutamate). The NPVF^804^ is to mimic the non-phosphorylatable tyrosine (Xu *et al.* 2010). NPVA^804^ is to abolish the tyrosine residue (Chen *et al.* 2006). NPVE^804^ is to mimic the phosphorylation (Qiu *et al.* 2019), with the expectation that three CRISPR engineered lines would display defective motility and abnormal cell migration. None of the lines, however, showed lethality or noticeable abnormal appearance, but they showed defective gonad migration (Figures 1B and 1D) and mild movement defects (Figure 1D). All alleles displayed a significant percentage of DTC migration defects (>30%) (Figure 1D). It should be noted that the DTC Mig was observed more frequently in the posterior gonad in *kq8041* (NPVA^804^) and *kq8042* (NPVE^804^), while the DTC Mig of *kq8043* (NPVF^804^) was equally detected in both gonad arms. All alleles showed the decrease in motility; the *kq8042* was severer than other alleles. We believe our results are useful for *in vivo* analysis of integrin functions and cell-matrix interactions.

## Methods

The CRISPR-Cas9 was used to edit the *pat-3* locus to create the *kq8041*, *kq8042*, and *kq8043* mutations. The potential crRNA sequence was present in exon 8 of the *pat-3* gene covering the membrane-distal NPVY (Figure 1C). The DNA repair template spans 48 bases upstream and 38 bases downstream of the target site, tyrosine (Y^804^). Synonymous mutations modified many positions of codons in the crRNA target to identify the mutation. The repair DNA templates, crRNA, tracrRNA, and Cas9 nuclease were custom made from IDT Inc., Coralville, IA. A mixture of template DNA (PAT3Y2A, PAT3Y2E, or PAT3Y2F), crRNA (ZQPAT3B), tracrRNA (cat. no. 1073190), and Cas9 protein (cat. no. 1081058) was annealed at room temperature. The mixture was micro-injected into the syncytial gonad of N2 animals (P0) with *dpy-10* co-CRISPR (Paix *et al.* 2015; Dickinson and Goldstein 2016). F1 animals with the Dpy phenotype were isolated, which displayed the mutation in single worm PCR genotyping with mutant specific primers (PCR-R-Y2F, PCR-R-Y2E, and PCR-R-Y2A) (Jansen *et al.* 1997). The Non-Dpy F2 homozygote was isolated; the animal displayed the mutation-specific PCR result but showed the absence of wild-type PCR result (the wild-type specific primer, PCR-WT-R-Y2). Homozygous mutants were crossed back to N2 two times. Three CRISPR edited mutant alleles, *kq8041* (NPVA), *kq8042* (NPVE), and *kq8043* (NPVF), were generated. Each edited line underwent phenotype analyses. Briefly, mutant animals showed DTC migration defects under a Nomarski microscopy (Lee and Cram 2009). Morphology of U-shaped gonad arms was observed in L4 or young adult stage hermaphrodites. For thrashing assays, animals were placed in 10 μl M9 drops. The number of body bending in aqueous solution was measured for 10 seconds. A Fisher’s Exact test (DTC migration) and Student *t*-test (motility assay) was performed to confirm the statistical significance of assay results. Nucleotide sequences of repair template, PCR primers, and crRNA in this study are listed below.

**Table:** 

**Repair template**	**Sequence (5’-3’)**
**Repair Y804F**	**GAGAACCCAATCTACAAACAGGCCACGACAACATTCAAGAACCCGGTT*TTT*GCAGGAAAAGCCAACTAAatagtttttatccttatatt**
**Repair Y804E**	**GAGAACCCAATCTACAAACAGGCCACGACAACATTCAAGAACCCGGTT*GAA*GCAGGAAAAGCCAACTAAatagtttttatccttatatt**
**Repair Y804A**	**GAGAACCCAATCTACAAACAGGCCACGACAACATTCAAGAACCCGGTT*GCT*GCAGGAAAAGCCAACTAAatagtttttatccttatatt**

Differentiated amino acids are italicized

**Table:** 

**PCR primers**	**Sequence (5’-3’)**	**Used for**
**PCR-WT-R-Y2**	**CCAGCGTATACTGGATTTTTA**	**wildtype specific**
**PCR-R-Y2F**	**GCAAAAACCGGGTTCTTG**	**Y804F specific**
**PCR-R-Y2E**	**CTTCAACCGGGTTCTTG**	**Y804E specific**
**PAT3MCRF**	**CATGATAGATCCGAATACGC**	**sequencing Forward**
**PCR-R-Y2A**	**CAGCAACCGGGTTCTTG**	**Y804A specific**
**PAT3R3UTR**	**acaatttatcgctaaatactcgtt**	**sequencing Reverse**

crRNA sequence

**Table:** 

**ZQPAT3B**	**5’-TTTAAAAATCCAGTATACGC-3’**	**TGG (PAM target)**

## Reagents

BU8041 *pat-3(kq8041)*, BU8042 *pat-3(kq8042)*, and BU8043 *pat-3(kq8043)* are available upon request.
